# Applicability and Design Considerations of Chaotic and Quantum Entropy Sources for Random Number Generation in IoT Devices

**DOI:** 10.3390/e27070726

**Published:** 2025-07-04

**Authors:** Wieslaw Marszalek, Michał Melosik, Mariusz Naumowicz, Przemysław Głowacki

**Affiliations:** 1 Department of Computer Science, Opole University of Technology, PL-45-758 Opole, Poland; 2 Institute of Computer Science, Faculty of Computer Science and Telecommunications, Piotrowo 3a, PL-60-965 Poznan, Poland; michal.melosik@put.poznan.pl (M.M.); mariusz.naumowicz@put.poznan.pl (M.N.); 3 Institute of Materials Research and Quantum Engineering, Faculty of Materials Engineering and Technical Physics, Poznan University of Technology, Piotrowo 3, PL-60-965 Poznan, Poland; przemyslaw.glowacki@put.poznan.pl; 4 Central Office of Measures, Elektoralna 2, PL-00-139 Warsaw, Poland

**Keywords:** IoT, chaotic generators, QRBG, Quantis QRNG, PRBG, chaos, quantum metrology, OpenLane, SkyWater PDK, CMOS, FPGA, ASIC

## Abstract

This article presents a comparative analysis of two types of generators of random sequences: one based on a discrete chaotic system being the logistic map, and the other being a commercial quantum random number generator QUANTIS-USB-4M. The results of the conducted analysis serve as a guide for selecting the type of generator that is more suited for a specific IoT solution, depending on the functional profile of the target application and the amount of random data required in the cryptographic process. This article discusses both the theoretical foundations of chaotic phenomena underlying the pseudorandom number generator based on the logistic map, as well as the theoretical principles of photon detection used in the quantum random number generators. A hardware IP Core implementing the logistic map was developed, suitable for direct implementation either as a standalone ASIC using the SkyWater PDK process or on an FPGA. The generated bitstreams from the implemented IP Core were evaluated for randomness. The analysis of the entropy levels and evaluation of randomness for both the logistic map and the quantum random number generator were performed using the *ent* tool and NIST test suite.

## 1. Introduction

### 1.1. General Consideration

Entropy sources are fundamental building blocks of random number generators used in electronic microsystems that perform cryptographic functions. In Internet of Things (IoT) solutions, beyond the registration, collection, and processing of data, a crucial aspect is to ensure the security of data. Consequently, it becomes necessary to select hardware cryptographic solutions that are adapted to the capabilities and limitations of devices operating within the IoT framework, as well as the constraints of the project’s budget. Data security is ensured both by the cryptographic algorithms used, and by the selection of an appropriate random number generator [1,2,3,4]. The random bitstream is used not only to create unique access passwords for device or system users, but it is also essential for generating initialization vectors in selected encryption modes for block cipher algorithms and in certain communication protocols [5,6,7]. Furthermore, the random bitstream is responsible for data security in the one-time-pad method [8,9]. Designing IoT devices that require data protection procedures often requires making decisions about which random number generator is more cost-effective and easier to integrate into the system.

A noticeable trend in recent scientific research has been the application of chaotic circuits in cryptographic systems. In continuous-time chaotic systems, analog signals are transformed into binary form using comparators or analog-to-digital converters. To ensure that the resulting sequence exhibits the necessary randomness properties, additional randomness extractors are often used, such as von Neumann correctors [10,11,12,13]. An electronic system, consisting of an analog chaotic circuit (as an entropy source), comparator, and randomness extractor, is considered a true random number generator, capable of producing a bitstream suitable for cryptographic processes. Beyond analog circuits for chaotic generators used in random bitstream generation, implementations based on optical chaos are also employed [14]. These solutions offer some of the highest random bitstream generation rates, reaching approximately 100 terabits per second. Despite the necessity of access to specialized laboratory infrastructure for such solutions, the high efficiency of this random bit generation process suggests that attempts might be made to integrate these types of generators into stationary solutions, for example, large data centers.

Discrete-time chaotic systems used as pseudorandom number generators have become much more popular in practical applications [15,16]. This popularity is primarily due to the ease of implementing discrete equations directly in digital form, for example, using hardware description languages like VHDL and other digital logic design techniques [17,18]. A random bitstream from a digital chaotic system can be obtained by extracting the fractional part of successive chaotic values, represented, for example, in fixed-point format (used to represent real numbers in digital systems) [19,20,21]. In such an approach, a randomness extractor is not always necessary. However, it can be noted that the unpredictability in such systems is only pseudorandom in nature, as it relies on the specification of the initial condition (treated as a seed) for the digitally implemented discrete chaotic equation. The use of discrete chaotic systems in a digital approach eliminates the need to design analog circuits and integrate them with the hardware layer on a dedicated circuit (see, for example [10]) or on specially designed PCBs. In the case of pseudorandom number generators, direct integration with the hardware layer of the designed systems is possible, as these systems often rely on FPGA platforms or dedicated ASICs. A high-entropy bitstream can also be provided to the system through other types of generators, such as quantum random number generators. Their operating principle is based on the detection of single photons passing through a semi-transparent mirror, which enables the generation of random outcomes in accordance with the fundamental principles of quantum mechanics. Depending on which of the two photon detectors registers the passage of a photon, a bit with a value of zero or one is added to the output vector forming the random bitstream. This process ensures a high level of unpredictability and high throughput in random data generation, making the quantum entropy sources particularly attractive for cryptographic applications. However, quantum entropy sources are not yet widely adopted in IoT systems.

So far, in research on the practical use of random number generators (based on chaotic or quantum phenomena), apart from analyzing entropy levels and evaluating randomness properties, there has been no attempt to conduct a different type of analysis that emphasizes the practicality and relevance of choosing a given solution for IoT. Entropy and randomness are key parameters in the context of cryptographic system security. However, for IoT systems, other factors must also be considered, particularly those related to the physical implementation and the intended functional purpose of the IoT devices.

### 1.2. Motivation

In this article, we present a comparative overview of chaotic and quantum sources of randomness for the purpose of generating random bitstreams in IoT applications. This overview is based on three criteria, two of which apply to both the true random and pseudorandom bitstream generation, while the third one addresses a challenge specific to the pseudorandom bitstream generation. The three criteria are as follows:

1. *Miniaturization and integration with the hardware layer of IoT devices*. This criterion reflects the importance of seamless hardware-level incorporation of the entropy source in the resource-constrained embedded systems.
2. *Level and performance of randomness source in IoT environments.* The rigorous evaluation of a randomness level and performance in IoT environment is absolutely critical because of the principles (foundation) of the cryptographic security. Without consistently maintaining strong entropy, IoT devices become highly susceptible to sophisticated attacks, leading potentially to data breaches, unauthorized access, and compromised system integrity. Therefore, assessing both the statistical randomness level and the rate at which these random numbers are created is essential to ensure the long-term resilience and trustworthiness of IoT deployments.
3. *Feasibility of implementing a reseeding mechanism (access to diverse seed values).* This aspect addresses the practical and architectural constraints associated with ensuring sufficient (high quality) entropy inputs during device operation. In particular, the article discusses the challenges posed by limited or intermittent access to high quality entropy sources, which can significantly impact the reliability and unpredictability of pseudorandom generators in real-world IoT deployments.

Based on the above three aspects, we propose a decision checklist, allowing a selection of an appropriate type of randomness source dedicated to IoT. The analysis is based on a specific discrete chaotic system and on a commercial quantum random number generator. For the purpose of this article, the logistic map equation was used for the digital implementation, and the selected quantum random number generator is the QUANTIS-USB-4M developed by the Swiss company ID Quantique.

## 2. Chaotic Logistic Map as a Source of Randomness for IoT

One of the simplest chaotic systems is the logistic map, described by a single nonlinear iterative equation in which each term of the generated sequence depends on the previous one [22,23]:

(1) $$x_{n+1}=rx_{n}\left(1-x_{n}\right),$$

where r>0 is the growth parameter and $x_{n}$ is the state variable for n=0,1,2,…, with $x_{0}$ being an initial condition.

**Figure 1:**
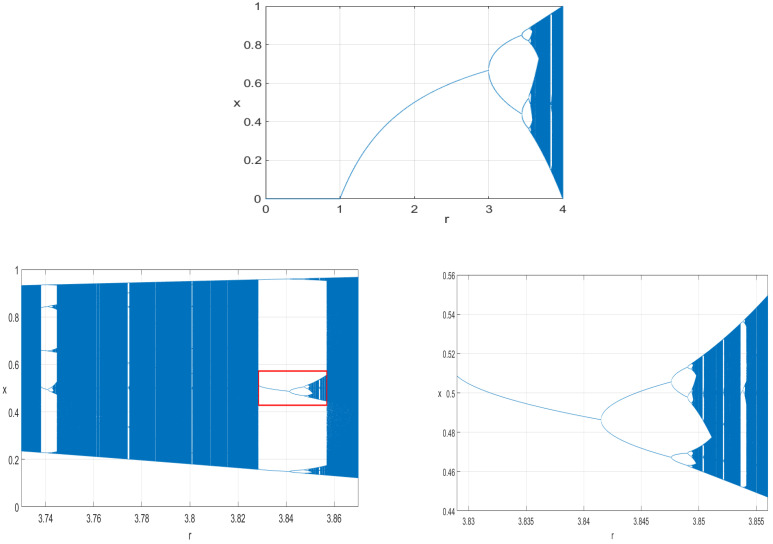
Bifurcation diagrams (local maximum values of $x_{n}$) in (1) for 0≤r≤4 (**top**), 3.73≤r≤3.87 (**bottom left**), and an enlarged view of the diagram in the red rectangle (**bottom right**). Windows of *r* with periodic and chaotic solutions are clearly recognizable.

The system’s behavior, attractors, time-domain response, bifurcation, and path to chaos are well known (see, for example, [24] for details). Periodic and chaotic solutions of (1) show interesting patterns with varying parameter *r*. Periodic windows in the bifurcation diagrams for the logistic map intermingle with chaotic windows, including period doubling roads to chaos and the sudden ceasing of chaotic attractors. An outline of the difference between the chaotic features and randomness is also presented there. An analysis of the logistic map’s behavior with varying *r* and various $x_{0}$ values is presented mathematically, for example, in [25]. Figure 1 shows a general bifurcation diagram of (1) for varying 0≤r≤4 (top), together with two specific diagrams extracted for the general diagram for narrower intervals of *r*. Equation (1) is suitable for direct digital implementation using FPGA devices, serving as pseudorandom generators, based on the phenomenon of deterministic chaos.

Digital IP Core logistic maps (or other discrete chaotic systems) can be used as pseudorandom generators or in applications as cryptographic modules used in encryption processes [26,27,28,29,30]. Most of the digital implementations of the logistic map described in the literature are suboptimal for the given hardware architecture. This is primarily due to the fact that such implementations do not use hardware description languages (HDLs) but rather rely on auxiliary tools like Simulink [31]. Nevertheless, these solutions are suitable for preliminary prototyping purposes or are utilized in the educational process. Generators designed in this way exhibit randomness characteristics, as confirmed by successfully passing NIST tests. Digital implementation using FPGA devices is just one of two approaches for physically constructing a digital system. The second, more power-efficient approach, and one that is better suited to a dedicated target application, is the implementation in an ASIC form. It should be noted that the implementations of logistic equations described in the literature using Simulink are not suitable for direct implementation as an ASIC-based integrated circuit. So far, the literature has not presented an example of how, using automated EDA design tools, the logistic map can be implemented in a form that is both synthesizable for a specific FPGA architecture and ready for the fabrication of a final ASIC integrated circuit in a selected CMOS process technology.

### 2.1. Integration of Chaotic Source

The hardware layer of IoT devices is often based on microcontrollers, FPGA devices, or ASICs. The choice of the second and third approaches allows for the integration of multiple modules within a single electronic system, such as communication interfaces, signal processing blocks, control algorithms, and encryption modules [32,33,34,35,36]. Each of these functionalities is typically implemented using independent IP Cores, available in several variants: commercial, open-source hardware through platforms like GitHub, or custom implementations developed by a project team. The flexibility of FPGA architectures and their relatively large hardware resources eliminate the need for multiple independent microelectronic systems for IoT, which would still have to be mounted and interconnected on a single PCB. In this way, the entire IoT device can be implemented in a single FPGA device. From a design perspective, this provides a significant advantage as there is no need to integrate an external source of randomness with the IoT hardware layer. In this approach, the randomness source or its selected components can be directly implemented within the hardware layer of the IoT device. This makes the prototyping process faster and more flexible. ASIC-based solutions fabricated in specific CMOS technologies are more optimal in terms of current and power consumption. For many years, licensing restrictions in accessing selected nanometer technologies (for use in industrial solutions) and the purchase of specialized EDA design tools made the prototyping process of ASICs expensive and extremely complex as well as time-consuming [37,38]. However, in recent years, there has been a development of open-source tools for automatic ASIC design. One of the most popular tools of this kind is OpenLane. It is an EDA tool used for automatic ASIC design and serves as an alternative to expensive commercial solutions [39,40,41]. This allows, via the HDL language for FPGA-typical implementations, for the preparation of a complete chip design together with a layout for a chosen CMOS technological process. In the past, such a migration required the development of proprietary EDA-type tools [42].

**Figure 2:**
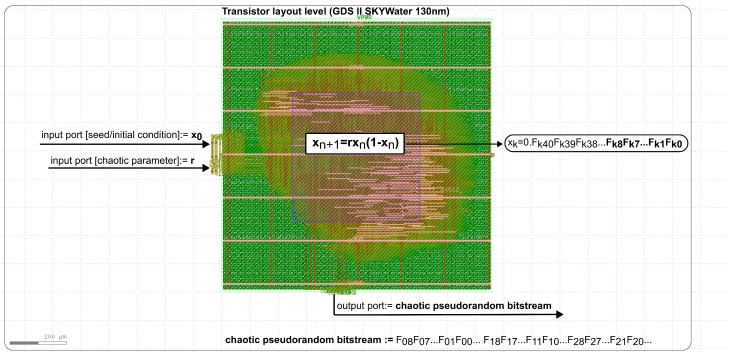
Layout of the IP Core intended for implementation in ASIC, generated on the basis of the HDL implementation and using OpenLane for the SkyWater PDK. The HDL source code was transformed into a transistor-level schematic, from which the layout shown in the figure was generated. The full source code of this implementation is available on GitHub [44].

The use of HDL in combination with OpenLane tools and SkyWater PDK technology makes ASICs functionally equivalent to FPGA-based implementations [43]. This results in the easy migration of the design to ASICs (from FPGAs) to be more optimal in terms of the chip power consumption, performance, and die area, while maintaining the original design intent. Most of the research known so far on the use of a logistic map as a pseudorandom generator focuses on its mathematical properties, neglecting the issue of how to design an IP Core that is suitable for integration into a specific CMOS process. An ASIC realization brings many benefits to IoT solutions. Among other things, it could allow the power consumption to be lowered in the context of typical FPGA-based solutions, if the device being designed is intended for mass production. The chip inside has a built-in independent source of pseudo-randomness obtained under conditions that guarantee the occurrence of chaotic dynamics. Such a design eliminates the need for external devices acting as a source of randomness. Figure 2 shows an example of an implementation of a logistic map equation in IP Core form for the SkyWater PDK 130 nm process, using the OpenLane tool. Table 1 presents a summary of the logic gates used in this implementation, along with the integrated circuit area and clock frequency. OpenLane offers a complete design flow, including the specification, synthesis, optimization, and generation of physical implementations of ASICs. Due to its flexibility and support for various process technologies (e.g., SkyWater PDK 130 nm), OpenLane can become an attractive choice for design teams working on IoT solutions. The ASIC (with logistic map IP Core) design process using OpenLane and HDL can be outlined in five stages, as follows:

1. **Hardware Description in HDL**—This step involves expressing the logistic equation using the HDL hardware description language. The HDL code defines the architecture of the digital system, including its input and output ports, registers storing the current state of the generator, and computational units performing calculations based on the mathematical formula.
2. **HDL Synthesis**—After creating the pseudorandom generator description in HDL, the next step is to synthesize it using the OpenLane tool. The synthesis process transforms the logical design description into a transistor-level schematic suitable for fabrication.
3. **Circuit Optimization**—The optimization phase aims to improve the system’s performance, including reducing the power consumption, shortening the computation time, and minimizing the chip area.
4. **Layout Generation**—Following optimization, OpenLane generates the ASIC layout, which is the physical representation of the logical design. For a pseudorandom generator based on the logistic equation, the layout includes the careful placement of logic components. It also adheres to technological constraints defined in the SkyWater PDK, which specify the design rules for element placement and routing in the fabrication process.
5. **Preparation for Fabrication**—At this stage, OpenLane performs additional verification steps such as Design Rule Check (DRC), Layout Versus Schematic (LVS), and Parasitic Extraction (PEX) to ensure that the layout complies with the technological requirements and is ready for manufacturing.

**Figure 3:**
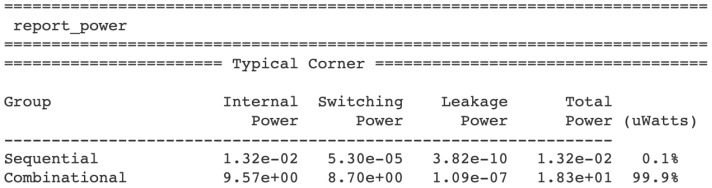
The power consumption analysis determined automatically across the three types. The integrated circuit’s package and its pads were not included in the simulation. The dominant characteristic of power consumption pertains to combinational logic.

In the ASIC design process, the pseudorandom number generator IP Core based on the logistic map (serving as a direct source of entropy) can be integrated with other IP Cores to create a dedicated hardware layer for IoT devices. This type of integration allows for the combination of various functions, such as pseudorandom number generation, data encryption, signal processing, or communication interfaces, to be combined into a single ASIC chip. Each IP Core can be responsible for a specific functionality, and their integration enables the creation of a complex yet cohesive solution. In the case of a pseudorandom generator based on the logistic map, its integration with other hardware components such as cryptographic modules (e.g., AES), communication interfaces, or signal processing algorithms allows for the creation of a complete and optimized custom hardware layer. As a result, the entire IoT device, including the functions related to random number generation, can be implemented within a single chip, leading to lower energy consumption, reduced production costs, and improved performance. The power report in Figure 3 reveals that the combinational logic overwhelmingly dominates, consuming 99.9% of the total power, primarily due to its internal and switching power factors. This is a direct consequence of the pipelined processing, where all logical and arithmetic blocks operate concurrently. The higher power consumption in the combinational section stems from the implementation of internal signals up to 134 bits wide, a necessity for achieving high precision in fixed-point calculations. Nevertheless, the data obtained in the report qualify the implemented circuit for typical battery-powered IoT applications. Given that OpenLane, along with the tools utilized in conjunction with it (e.g., ngspice), are open source and thus not free of bugs, PVT analysis was omitted. Its execution is not critical for our implementation, unlike in certain cases, where the circuits employing Physically Unclonable Functions (PUFs) are being designed.

### 2.2. Randomness Quality and Performance of Chaotic Source

For the designed IP Core presented in Figure 2, bits from successive values of x(n), (=$x_{n}$ in (1)) of the logistic map equation were recorded to create one long bitstream, which was then subjected to statistical analysis using the NIST statistical test suite. These tests are available as a ready-to-use software package developed and provided by NIST [45]. A high level of randomness is generally understood to equate to successfully passing almost all NIST tests. The more the NIST tests indicate problems in detecting randomness properties, the greater the observed degradation and the poorer the quality of the randomness. All deterministic random sources are characterized by periodicity after, possibly, a long transition interval, including generators based on the logistic map. Ideally, for real numbers with no limitation on the number of fractional places, the logistic map will not exhibit repeating consecutive values. However, when implemented, e.g., in fixed-point notation, periodicity may occur. A common compromise to extend the length of the random sequence is to increase the number of bits used to represent the real value of consecutive samples x(n) from the logistic map equation. A detailed analysis is presented in [26]. The issue of randomness degradation stemming from the digital implementation of chaotic systems, particularly in the context of induced periodicity, has been extensively discussed in the literature over the years [46,47,48]. Certain examples for such scenarios are shown in Table 2. For the first volume of data (10 sequences of 1 mln bits each), the minimum pass rate for each statistical test is approximately equal to 8 sequences for all 10 binary sequences. For the second volume of data (100 sequences of 1 mln bits each), the minimum pass rate for each statistical test, with the exception of the random excursion (variant) test, is approximately equal to 96 sequences for all 100 binary sequences. The minimum pass rate for the random excursion (variant) test is approximately equal to 62 sequences for a total of 66 binary sequences. For the third volume of data (10 sequences of 10 mln bits each), the minimum pass rate for each statistical test, with the exception of the random excursion (variant) test, is approximately equal to 8 sequences for 10 binary sequences. The minimum pass rate for the random excursion (variant) test is approximately equal to 8 out of 9 binary sequences. Table 2 was prepared based on an automatically generated summary report for the NIST tests. The NIST suite automatically determines the final number of sequences to be analyzed based on the user-defined parameters. For the Non-overlapping Templates, Random Excursions, and Random Excursions Variant tests, numerous sub-tests are executed. Their total count has been tallied and presented in a single row in the table. In Table 2, the red color is used to additionally indicate those tests for which the NIST suite, beyond analyzing the pass proportions, also pointed to a randomness issue in terms of the *p*-value. Increasing the amount of generated data from 10 million bits to 100 million bits (for both variants, that is, 100 sequences of 1 million bits each, and 10 sequences of 10 million bits each) demonstrates the sensitivity to NIST tests. When the number of bits increases, the statistical tests begin to detect randomness issues, caused by the representation of the real value $x_{n}$ as a finite-precision arithmetic representation.

For the automatic evaluation of entropy levels in both the pseudorandom and truly random generators, the most commonly used tool is the *ent* test [49,50]. This tool computes randomness metrics in the analyzed data based on information theory. The program measures the degree of unpredictability in a data stream, which can be treated as a series of bits or bytes. It is important to emphasize, however, that for large volumes of data containing random bits, the accuracy of the *ent* tool is not as high as the NIST statistical tests. A high entropy value indicates a high level of randomness and difficulty in predicting subsequent elements of the sequence. If a sequence is perfectly random, the entropy should reach its maximum value (8 bits per byte). Otherwise, a lower entropy value indicates a more predictable or correlated data stream. The entropy and basic randomness measurements for the logistic map and QUANTIS-USB-4M generator are shown in Table 3. It is important to note here a rather obvious but often overlooked fact in discussions regarding the design and testing of random number generators. A high level of entropy does not guarantee that a given sequence is truly random. The analysis of a sequence, whether from the perspective of entropy itself or other statistical tests, is not an absolute arbiter of whether a given generator (and bitstream) is truly random. The results indicating a high level of entropy and successful passes of statistical tests only confirm that the specific data segment, generated under precisely defined measurement conditions, exhibits statistical properties. In the context of hardware attacks, such as those employing hardware Trojans, an intriguing scenario—hitherto undescribed in the literature—is possible. During the generation of a random sequence for testing, a hardware Trojan integrated with the random bit generator remains inactive. The results of a bitstream analysis show its random characteristics. However, when the generator operates at its intended destination, the hardware Trojan changes the generator’s behavior, thereby affecting its level of randomness. This problem was first analyzed in [51]. The same problem also applies to quantum random number generators. Contemporary hardware threats underscore the long-established principle that randomness cannot be proven; it can only be refuted.

To eliminate the loss of the measurement’s values caused by generating packets with a random bitstream that is not actually being used by the system at a given instant, the pseudorandom number generator must operate in a request–response mode. The IoT system sends a request that triggers the generator to return a random value. Otherwise, when the generator operates in a continuous configuration, it may quickly reach periodicity by producing a non-random bitstream that is currently present within the system. Therefore, it becomes necessary to extend the IP Core with functionality that enables a request–response operation. Designing a system that controls the pseudorandom generator in a request–response configuration allows for a realistic performance assessment, taking into account actual requests from the system. This approach enables an extension of the interval between reseeding operations. For the designed IP Core, we are able to obtain 45 714 285 714 pseudorandom bits in 2 min of continuous operation (for clk freq = 47,619 MHz). If we assume that, in this time, the IoT system only needs to obtain two random vectors of 256 bits each, then the remaining bits from the generated sequence are not used and we waste the values that could be used in the next instant when such a need arises. It should be emphasized that the problem of wasted and unused random values from the pseudorandom generators has not been addressed so far in the studies of obtained and expected levels of randomness. However, such an analysis should take place when the full specifics of the IoT system into which the designed IP Core will be integrated are known. The ability to control a pseudorandom generator, understood as returning a random value in a request–response system, allows for the calculation of its performance. An excess of unused random bits does not add any value to the functioning properties of the system. In IoT systems, where energy efficiency plays a crucial role (e.g., in battery-powered devices), unnecessary activity of the generator becomes a significant issue. Each iteration of the logistic map requires the operations of multiplication and addition, which, in digital circuits, translates into increased energy consumption. By extending the IP Core with this type of additional circuitry (such as, for example, a request–response control unit), we extend its practical usefulness, without unnecessarily wasting (not using) further values that accelerate the effect of the appearance of periodicity in such a generator. Similar approaches are used in cryptographically secure random generators, such as, for example, Fortuna; see [52].

Excessive data generation that does not match the actual needs, along with increased energy consumption, makes IP Cores based on the logistic map potentially inefficient for IoT applications. The issue of the loss and underutilization of random values from pseudorandom generators is often overlooked in studies evaluating the performance of randomness sources based on deterministic algorithms. The dominant method for assessing the usefulness of a pseudorandom source relies solely on the analysis of NIST test results. What is frequently omitted is a discussion of how many random bits the generator must deliver to the cryptographic system and over what time span. Such an analysis should be conducted when the full specification of the IoT system—into which the designed IP Core will be integrated—is known. Designing a system that controls the pseudorandom generator in a request-based configuration allows for a realistic performance assessment, taking into account actual requests from the system. This approach enables an extension of the interval between reseeding operations.

### 2.3. Access to Diverse Seed Values

In deterministic random generators, periodicity occurs after all internal states have been traversed for a given seed value. For this reason, NIST recommends that pseudorandom number generators include an integrated reseeding mechanism when designing deterministic generators [53]. This recommendation also applies to generators based on the logistic map, where the seed value is $x_{0}$ in the logistic equation. However, this recommendation is often not taken into account when implementing the logistic map digitally as a pseudorandom generator [19,26]. Typical approaches aimed at guaranteeing security are limited to selecting the parameter $x_{0}$ at the implementation stage, without undertaking the implementation of a more complex IP Core that fulfils the aforementioned recommendations regarding the construction of deterministic sources of randomness. It should be emphasized that such a simplification in the selection of $x_{0}$ is permissible only for research or educational purposes, where we are dealing with demonstrative prototyping of the “proof of concept” type. For commercial deployment, such an implementation must be extended precisely with an IP Core featuring a reseeding mechanism. Instead of designing a reseed IP Core, a commonly preferred approach involves increasing the number of bits used to represent the fractional part of the real numbers, which is a questionable compromise. The system becomes vulnerable to brute-force attacks. Instead of searching the entire space of keys generated for multiple initial values, the attacker potential may focus on passwords generated from a specific initial value $x_{0}$. To avoid the occurrence of periodicity in the logistic map, it is necessary to implement a reseeding mechanism in accordance with the NIST guidelines for designing deterministic random number generators [53]. The present study deliberately omits the implementation of a reseeding mechanism. This choice was made to highlight the critical necessity of reseeding in real-world deployments and to unequivocally demonstrate the consequences of overlooking the specific design requirements for deterministic entropy sources. Our simulations and analyses clearly illustrate that this approach—characterized by the absence of a complex reseeding mechanism coupled with an increased number of bits recorded from the logistic map IP Core—leads to a measurable degradation of randomness, as evidenced by NIST statistical tests, which intensifies with the growing number of generated bits. As the IoT market continues its rapid expansion, the design of sophisticated reseeding mechanisms will likely evolve into a distinct research area, increasingly intertwined with the advancement and integration of external sensors. In the case of implementing the logistic map, this requires the development of a dedicated IP Core, responsible for controlling and managing the reseeding process. Its task is to cyclically replace the initial value $x_{0}$ after the generator has cycled through all states. Such functional separation of the two IP Cores, where the reseeding mechanism is implemented as a separate, independent IP Core, offers several benefits in the context of IoT devices. It becomes easier to verify and optimize each IP Core individually, which is crucial in the ASIC design process, where each system component must function according to specific requirements and standards. Such separation will make the design process more flexible, allowing for the later integration of both logic blocks in a way that best meets the specific needs of the IoT system. Having a separate IP Core for the reseeding mechanism provides greater control over its implementation, allowing for a more precise adaptation of the reseeding strategy to the specific requirements of the application and the external environment in which the device will be deployed. Due to the limited access to highly unpredictable seed values in ASIC systems, ensuring access to highly variable seeds from the environment becomes a difficult challenge that directly affects cryptographic security.

## 3. Quantum Phenomena as Sources of Randomness for IoT

**Figure 4:**
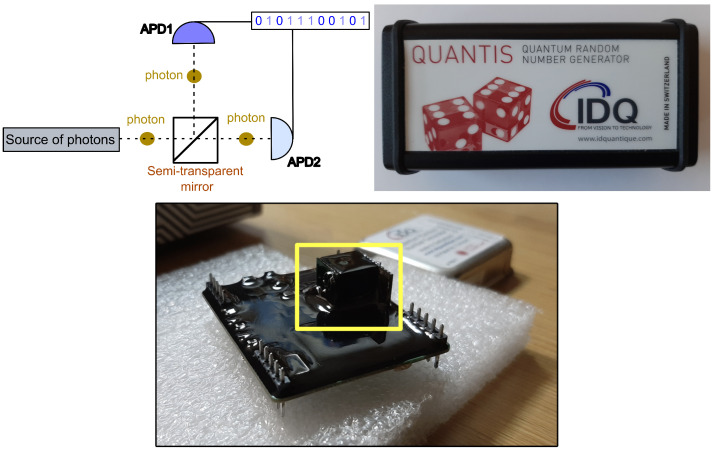
Illustration of the quantum random bit generator (QRBG) principle, employing dual photon detectors and a beam splitter (top left). This design is implemented in the commercial QUANTIS-USB-4M quantum random number generator, shown in the top right. The main electronic module responsible for quantum random bitstream generation is depicted below. The beam splitter (marked as semi-transparent mirror on top left of the schematic) with associated APDs are highlighted with a yellow border.

The risks in the cryptographic process arising from the repetition of random sequences by pseudorandom generators have created the need to seek new solutions that ensure the generation of truly random sequences-independent of initial conditions and the method of physical implementation. For this reason, there is a growing interest among IoT system designers in exploring the potential use of quantum random number generators. Quantum random number generators are no longer limited to research conducted exclusively in specialized laboratories. Their operation and the recording of random values are often handled via a USB interface. One example is the QUANTIS-USB-4M generator from the Swiss company ID Quantique [54]. The most common quantum phenomenon enabling the generation of a truly random bitstream is photon beam splitting. This setup requires a beam splitter and two photon detectors, such as APD diodes. A light source, typically from an LED or a dedicated laser, is directed to the input of the beam splitter. Photons pass through the beam splitter in a random manner, thus ensuring a 50% probability of being detected by either of the two photon detectors, as illustrated in Figure 4. Depending on which APD-based detector registers the photon, a bit value of 0 or 1 is added to the register forming a bitstream [55,56].

### 3.1. Single-Photon Avalanche Diodes (SPADs)

A Single-Photon Avalanche Diode (SPAD) is a type of avalanche photodiode operating in the so-called Geiger mode. A SPAD consists of two key elements:

- A high-electric field region in the *p-n* junction (obtained by the application of a high reverse bias voltage, exceeding the so-called breakdown voltage $V_{BD}$), where the creation of an electron–hole pair by a single photon incident on the photosensitive device area can trigger an avalanche of secondary carriers (through impact ionization, i.e., collision of the primary carriers with the lattice atoms); this results in a divergent (avalanche) multiplication.
- An external circuit, which quenches the discharge, i.e., stops the avalanche current.

In the Geiger mode, the avalanche current is self-sustaining. The parameters of the quenching circuit regulate the height of the pulse following the detection of a single photon.

**Figure 5:**
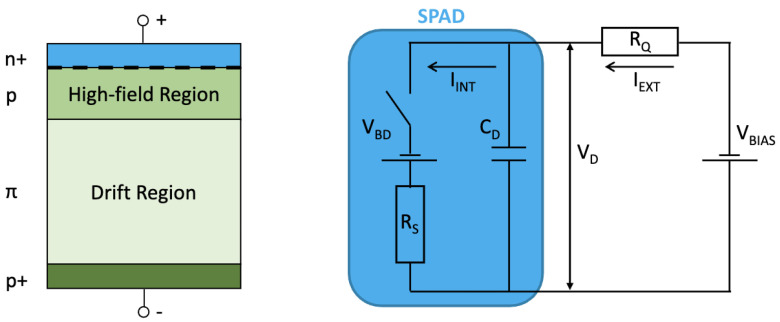
(**Left**) One-dimensional sketch of a possible doping structure (n+/p) of a SPAD. Junction is highlighted with a dashed line. (**Right**) Basic electrical model of a SPAD with a resistor $R_{Q}$ as the quenching element.

The SPAD structure was proposed for the detection of single photons in [57]. This structure is usually formed by an abrupt junction (n+/p or p+/n). For example, the structure of the n+/p junction is presented in Figure 5 (left).

The *p*-side includes a weakly doped layer (π) and an enrichment box (p). Reverse bias voltage causes the full depletion of the *p*-side and formation of the specific electric field distribution. The top part (where the electric field is high) constitutes the multiplication region, while the bottom part is the drift region, required for the photo-electrons to drift towards the high-field region. If the bias voltage exceeds the breakdown value, an avalanche is triggered when the carrier crosses the electric field peak at the border of the p and n+ layers.

The operation of the SPAD can be modelled by an equivalent electric circuit, depicted in Figure 5 (right). The main building blocks of the SPAD equivalent circuit are as follows: the junction capacitance $C_{D}$ at the operation voltage $V_{D}$, the resistance $R_{S}$ of the space charge at the avalanche region, a voltage source set at a breakdown voltage value $V_{BD}$, and a switch. The external elements comprise a bias voltage $V_{BIAS}$ supply and a series resistor $R_{Q}$. The current flowing through the switch in the SPAD is denoted as $I_{INT}$, while $I_{EXT}$ is the current in the external circuit.

The model of the SPAD assumes two probabilities that control the switch operation: an avalanche breakdown triggering probability $P_{T}$ and a quenching probability $P_{Q}$.

SPADs can be combined in arrays to form the so-called silicon photomultipliers (SiPMs). The main parameters of such devices are as follows:

1. **Breakdown voltage $V_{\mathrm{BD}}$**
  As already indicated, this parameter denotes the lower limit of the bias voltage, exceeding which brings the diode to operate in Geiger’s mode. The factors important in the applications include the $V_{BD}$ value and temperature dependence, as well as uniformity for the sets of individual devices.
2. **Photodetection efficiency (PDE)**
  This is the ratio of the number of photons detected and the total number of the photons incident on the device. In order to facilitate our analysis of the impact of various parameters, the PDE can be factorized as follows:
  (2) $$PDE\left(V;\lambda \right)=QE\left(\lambda \right)\times P_{T}\left(V,\lambda \right)\times FF.$$
  The indicated parameters depend on the wavelength λ and the bias voltage *V*. The QE denotes the quantum efficiency (the probability of the creation of an electron–hole pair by the absorption of an incident photon), FF is the SiPM cell fill factor, i.e., the ratio of the active area to the total area of the cell, and $P_{T}$ is the avalanche breakdown triggering probability (also referred to as the Geiger discharge probability).
3. **Primary and correlated noise**
  There are two main relevant categories of noise: primary noise (not related to photon absorption, but to accidental carrier creation by, e.g., thermal excitation, which is followed by an avalanche event), and correlated noise (an avalanche event generated as a consequence of a preceding avalanche event, regardless of its origin. The most important parameters include the following:
  - Primary Dark Count Rate (**DCR** [counts per second, cps]).
  - Primary Dark Count Rate dependence on temperature.
  - Optical cross-talk (emission of the so-called “cross-talk” photons by the hot carriers passing through the high-field zone).
  - Afterpulsing (second avalanches triggered occasionally by carriers captured by trapping centers in the high-field zone and then re-emitted).
  - Optically induced afterpulsing (afterpulsing caused by cross-talking photons).
4. **Timing properties**
  A relevant measure of the timing performance is the Single-Photon Time Resolution (SPTR). This parameter denotes the precision of the estimate of the time of arrival for a photon in the visible spectral region, incident on the detector surface at a random position. This timing parameter is obviously dependent on the characteristics of the detector itself, but also on the read-out.

A comprehensive description of the structure, operation, parameters, and applications of SPADs (and SiPMs) can be found, e.g., in the review article [58].

### 3.2. Photon Statistics

Perfectly coherent light with a constant intensity exhibits **Poissonian** photon statistics. This is described in detail in some books, e.g., [59,60].

The detection of a light beam by a photon counter, i.e., a very sensitive light detector, such as PMT or APD, connected to an electronic counter, should, in principle, provide information on the photon statistics. However, the issue of the identification of the photon statistics on the basis of photon detection is more complex. There are two aspects that have to be distinguished:

1. The intrinsic photon statistics of the light beam.
2. The statistical nature of the photodetection process.

This means that some detection events may be artefacts of the detection process, and are not directly related to the photon statistics. In an experiment, the number of photons incident on the detector in some specified time interval should be counted. For a perfectly coherent monochromatic beam with constant intensity *I* and frequency ω, the photon flux Φ (defined as the average number of photons at the beam cross-section in unit time) can be determined as follows:

(3) $$\Phi =\frac{IA}{\hbar \omega }=\frac{P}{\hbar \omega }\;\mathrm{photons}\times s^{-1},$$

where *A* is the beam cross-section and *P* denotes the power; ℏ=h/(2π) is the reduced Planck constant; and *h* denotes the Planck constant.

The basic characteristic of the photon counters is their quantum efficiency η, defined as the ratio of the number of recorded photons to the number of incident photons. The average number of photocounts registered in a time interval *T* is expressed as

(4) $$N\left(T\right)=\eta \Phi T=\frac{\eta PT}{\hbar \omega },$$

and the average **count rate** R is calculated as

(5) $$R=\frac{N}{\hbar \omega }=\eta \Phi =\frac{\eta P}{\hbar \omega }\;\mathrm{counts}\;s^{-1}.$$

The relationship between the variance in the photocount number ${\left(\Delta N\right)}^{2}$ recorded in time *T* and the corresponding variance ${\left(\Delta n\right)}^{2}$ in the number of photons incident on the detector during the same time interval, is given by

(6) $${\left(\Delta N\right)}^{2}=\eta^{2}{\left(\Delta n\right)}^{2}+\eta \left(\eta -1\right)\bar{n}.$$

In the above equation η denotes the quantum efficiency of the detector $\left(\eta =\bar{N}/\bar{n}\right)$, and $\bar{N}$ and $\bar{n}$ are the average photocount number and the mean incident photon number, respectively.

From (6), certain conclusions can be drawn, as follows:

1. For ideal quantum efficiency η=1, we have ΔN=Δn, which represents a strict correlation between the fluctuations of photocounts and incident photons.
2. For Poissonian statistics of the incident light beam, the following relation holds, independent of η: ${\left(\Delta n\right)}^{2}=\bar{n}$; ${\left(\Delta N\right)}^{2}=\eta \bar{n}\equiv \bar{N}$. This means that the photocount statistics are also Poissonian.
3. For the limiting case of a very low quantum efficiency η≪1, the photocount variance ${\left(\Delta N\right)}^{2}=\eta \bar{n}\equiv \bar{N}$, i.e., the photocount statistics are Poissonian, irrespective of the underlying photon statistics.

The last conclusion means that the determination of the actual photon statistics can only be reliably attempted with the use of detectors characterized by a high quantum efficiency. Then, the photocount statistics adequately reflect the photon statistics. The quantum efficiency is the key parameter characterizing single-photon counting devices. There are numerous types of single-photon sources, including NV^−^ color centers in diamonds [61,62]. It might be interesting to check if such a source entropy can be manipulated by temperature; it is well known that temperature changes impact various properties of this type of point defect (for example, [63,64,65]). Photodetectors (SPADs or SiPMs) are widely used in the detection of various types of optical signals, and also in devices that can be used for the ultra-precise time synchronization of various processes in IoT, i.e., atomic clocks, primary atomic standards [66], secondary optical atomic standards [67], or currently developed optical nuclear standards [68,69].

### 3.3. Integration of Quantum Source

The use of a self-built quantum randomness source (instead of a commercial one) in IoT applications requires the allocation of additional space in the designed device for analog components, which may occupy several cubic centimeters. The two APD diodes, the beam splitter, and the light source must be external components that will then be mechanically mounted on the PCB containing the entire IoT system. Figure 4 shows the main electronic module of the Quantis generator. The electronic circuit is surface-protected by a producer with dedicated resin, which prevents hardware attacks involving direct manipulation of the printed circuit. The yellow square indicates the beam splitter used to split the photon beam. It is possible to miniaturize such a structure, but under the standard conditions of an electrical laboratory, full integration with both FPGAs and ASICs is extremely difficult. The approximate dimensions of such a setup, as implemented in the QUANTIS-USB-4M generator, are 10–15 mm × 10 mm × 10 mm. The entire system can be operated using a selected microcontroller or an FPGA, which is responsible for inserting 0s and 1s into a shift register to incrementally and continuously generate a random bitstream to be used later for cryptographic purposes. If one decides to build an analogue quantum circuit on their own and further integrate it on a PCB, a second significant problem arises. Such a circuit with free access from the PCB surface poses the risk of making unauthorized modifications to its structure or introducing additional subcircuits as hardware Trojans that could hack its functionality. It remains an open question whether such surface protection of the electronic circuit with resin (as is the case with IDQuantique) guarantees complete resistance to hardware Trojan attacks classified as being introduced into the circuit at the assembly/testing level [70,71,72,73,74]. Such a risk has been discussed in the context of chaotic generators in the form of PCB-mounted analogue circuits [51]. The same threat cannot be excluded in the context of self-constructed quantum random generators operating in an unknown environment. For the time being, the safest possible integration of quantum random number generators is the use of commercially available devices, e.g., from the company IDQuantique (QUANTIS-USB-4M generator), which offers a quantum generator with a USB interface. The use of this type of solution entails an increase in the power consumption of the IoT device being designed, while significantly increasing its size. To obtain random data via the QUANTIS quantum generator, it is necessary to use dedicated software supplied free of charge by the manufacturer. This software requires a Windows or Linux operating system. Such an approach is significantly more problematic to be completed in the context of IoT systems, as its hardware layer (in some cases) should allow the operating system to run on one of the popular SBC devices.

### 3.4. Randomness Quality and Performance of Quantum Source

Due to the quantum nature of the phenomenon used for randomness generation, quantum random generators always show positive results for almost all NIST randomness tests. Since the QUANTIS-USB-4M relies on a mechanism based on physical quantum processes, its randomness does not exhibit deterministic patterns or periodicity, which are characteristic of pseudorandom generators. As a result, even when analyzing large volumes of data, the generated bitstream meets the statistical requirements of tests such as those from the NIST suite. Prior to being made available to the end-user, the bits can undergo a post-processing method, consistent with ID Quantique’s implementation detailed in their technical report [75]. Once available to the user, these bitstreams are typically subjected to randomness analysis by the user, for instance, using the NIST Statistical Test Suite. The results of the NIST tests used to evaluate the randomness level of the quantum generator are presented in Table 4. The NIST tests’ reports are as follows:

For the first volume of data (10 sequences of 1 mln bits each), the minimum pass rate for each statistical test, with the exception of the random excursion (variant) test, is approximately equal to 8 sequences for all 10 binary sequences. The minimum pass rate for the random excursion (variant) test is approximately equal to 4 sequences for 5 binary sequences. For the second volume of data (100 sequences of 1 mln bits each), the minimum pass rate for each statistical test, with the exception of the random excursion (variant) test, is approximately equal to 96 sequences for all 100 binary sequences. The minimum pass rate for the random excursion (variant) test is approximately equal to 48 sequences for a sample size of 51 binary sequences. For the third volume of data (10 sequences of 10 mln bits each), the minimum pass rate for each statistical test, with the exception of the random excursion (variant) test, is approximately 8 sequences for all 10 binary sequences. The minimum pass rate for the random excursion (variant) test is approximately equal to 7 sequences for all 8 binary sequences. Similarly, as in Table 2, for the Non-Overlapping Templates, Random Excursions, and Random Excursions Variant tests, multiple sub-tests were aggregated and counted within a single row. Both the pass rate for proportions and the *p*-value showed no issues with randomness. The amount of bits returned by the quantum generator does not affect the randomness disturbances in the analyzed bit sequences.

Across more than two decades of scientific literature, a discussion on the term randomness and, in particular, “true randomness”, appears to be a hot topic. The general agreement holds that if a bitstream passes NIST tests and is not derived from a deterministic pseudorandom algorithm, it can be considered a binary sequence from a true random source. The question of whether “true randomness” can be legitimately asserted is explored, for example, in [76].

The NIST tests do not verify the intrinsic randomness of the underlying physical phenomenon upon which a QRNG is constructed. The tests merely ascertain whether the recorded sequence exhibits random characteristics as defined by a series of statistical tests. These tests do not examine the generator’s resilience, as a device, against potential failures or its susceptibility to hardware attacks. Consequently, doubts may arise as to whether a given sequence can be termed “truly random” or merely “exhibiting random properties”. A comprehensive discussion on this topic would require a broad, interdisciplinary examination of the validity of using the term “truly random,” especially in the context of CHSH [77]. The violation of the CHSH inequality is a key method for confirming randomness of QRNGs. As was stated in [78] by demonstrating that the correlations between measurement outcomes violate the CHSH inequality, it is possible to certify that the generated random numbers are “truly random” and not predictable, even with full knowledge of the device’s internal workings. Device-independent quantum random-number generation (DIQRNG) based on the loophole-free violation of a Bell inequality produces genuine “unpredictable randomness” without requiring any assumptions about the inner workings of the devices [78].

The QUANTIS-USB-4M generator allows bitstream to be achieved at 4 Mb/s. This generator can operate continuously without the risk of exposing the cryptographic process to potential periodicity. The Quantis generator generated 480 million random bits in 2 min. The Quantum random bits generator does not necessarily have to work in a request–response system, but this may make sense if we want to save battery power. Such an approach enables the unlimited generation of vectors of varying lengths for cryptographic functions in IoT, dynamically adapting to the system’s demand. As a result, this allows for the efficient use of randomness resources, depending on the requirements of a specific cryptographic operation—regardless of whether a single random value or a large bitstream is needed. However, a high capacity for random bit generation can impose an additional burden on the system concerning data acquisition, processing, and transmission. Consequently, practical implementations may necessitate the development of mechanisms for storing random data and utilizing it according to the specific requirements of the IoT applications. The generator’s manufacturer ensures the device is resilient to environmental changes when operating under typical ambient conditions. However, as demonstrated in [79], temperature variations can impact the level of randomness in the generated bitstreams. The influence of temperature on randomness degradation is determined by the particular electronic components, such as the type of diodes, employed in the quantum random number generator’s design. For the QUANTIS-USB-4M generator, its main module (highlighted in yellow in Figure 4) is secured with a coating, over which a metal shield is applied, and the entire assembly is enclosed in a compact casing. This design ensures that, under standard operating conditions, the influence of typical ambient temperature should not affect the generator’s performance. However, in the context of hardware Trojan attacks, Side Channel Attacks, and in conjunction with the results described in [51], the issue of temperature’s influence on the performance of quantum random number generators (especially commercial ones) should be further investigated in independent studies.

## 4. Entropy Source Selection for IoT Devices

**Figure 6:**
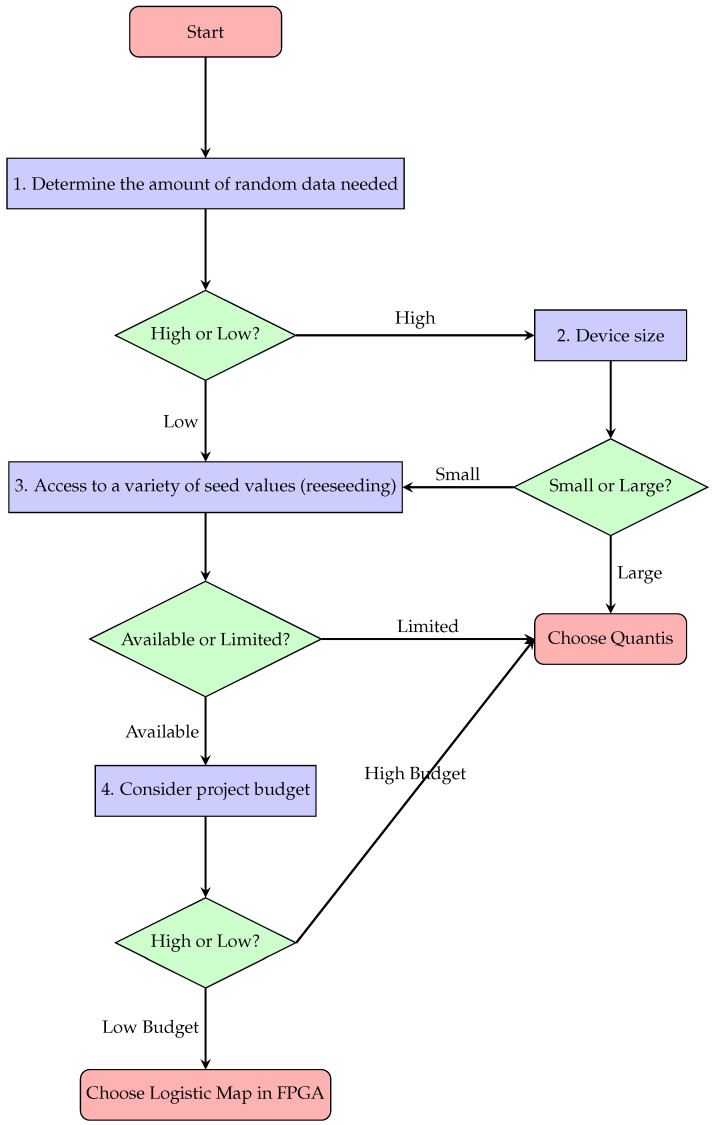
Decision tree guiding the selection of a randomness source (pseudorandom logistic map generator or QUANTIS-USB-4M quantum random bit generator) for IoT devices, based on an analysis of four key factors: required random data volume, device dimensions, reseeding capabilities, and available budget.

To enhance the transparency and facilitate the selection process of a suitable randomness source for IoT systems, particularly for electronic system designers not specializing in cryptographic device design, we propose a decision checklist shown in Figure 6. This checklist enables the selection of a more appropriate generator type for a given IoT solution. The development of this checklist involved the identification of four key factors that play a crucial role in the design of IoT systems, requiring the integration of a random number generator for security: the actual **volume of random data** that must be supplied to the system to ensure its security; **the physical dimensions** of the intended device; the accessibility of **highly diverse seed values**; and the **available budget** for device design. The parameters for the decision tree shown in Figure 6 are presented in Table 5. The threshold values were selected based on the authors’ own experience in designing FPGA devices and integrated circuits [38,52]. The provided reference values are illustrative examples and pertain to an IoT device implementation that does not require processing large amounts of data. The selection of these parameters will depend on the specifics of the target solution, primarily on the volume of data the device will process and its data transmission speed. These requirements will dictate how frequently the cryptographic algorithm will need to fetch new values from the random number generator. More extensive research into appropriate reference values is warranted and could lay the groundwork for future in-depth studies. Such studies would focus on optimizing the efficiency of generated random bitstreams and ensuring their full practical utilization across various IoT scenarios. For devices designed to process only a few data points with infrequent remote transmission, the amount of random data needed for cryptographic purposes is limited, making generators with a lower bitstream generation throughput preferable. For devices with moderate enclosure sizes, the integration of supplementary quantum hardware, including commercial quantum random number generators, is a feasible but not an easy implementable option. However, if the designed device must adhere to strict size constraints, the selection of pseudorandom number generators, which offer more complex system integration (in the sense of configuration and specifically used EDA tools), becomes necessary. A critical aspect to consider when selecting a random number generator type is the accessibility of highly diverse seed values. While the implementation of a reseeding mechanism may not present a significant design challenge, a situation can arise where the operational specifics of the IoT device within its intended deployment environment hinder the provision of highly variable seed values to this mechanism. In such circumstances, it becomes necessary to exert effort to modify the device’s design specifications to accommodate the integration of a commercial quantum random number generator, if the physical dimensions allow. Failure to do so risks the occurrence of the periodicity phenomenon discussed earlier, thereby posing a threat to the entire cryptographic process. If the environment in which the IoT device will be deployed facilitates the provision of diverse seed values, thus ensuring the security of the reseeding process, a key consideration is the available budget for the device design. Unconstrained access to financial resources during the design and implementation phases permits a re-evaluation of the design approach, potentially allowing for a larger device with SBC integration and a reliable, stable power source, thus making the utilization of commercial quantum random number generators the best fit.

As depicted in the decision checklist presented in Figure 6, the selection of a randomness source type is contingent upon the four factors previously discussed. These factors are intrinsically linked to the application-specific requirements and operational demands of each individual device. Quantum random number generators offer truly random values, eliminating the periodicity issues inherent in chaotic solutions such as the logistic map. Their primary advantage lies in their capacity to generate an unlimited number of random vectors in a continuous manner, significantly enhancing the entropy levels. However, their implementation necessitates dedicated hardware, including photon detection circuits and a supplementary SBC for managing the bitstream recording software. This translates to a larger PCB footprint and increased integration complexity compared to purely digital solutions.

Conversely, generators based on the logistic map can be directly implemented within FPGAs or ASICs, rendering them more flexible in terms of hardware integration. Nevertheless, their deterministic nature necessitates an additional reseeding mechanism to mitigate the predictability of the generated values. The ultimate choice between these technologies hinges on the specific deployment context and the quantity of random data required. In systems where security and unpredictability are paramount, quantum random number generators offer the highest level of protection at the expense of more demanding hardware requirements. In contrast, for IoT applications where energy efficiency and integration with existing infrastructure are prioritized, the logistic map can represent a practical trade-off, provided that the reseeding mechanism is effectively managed. Failure to adequately address the reseeding mechanism during the implementation phase can have catastrophic consequences for the cryptographic security process.

## 5. Conclusions

In this article, we present a comprehensive overview of the challenges associated with generating randomness in the context of the constraints and requirements imposed on IoT solutions. In contrast to the majority of existing research results, which focus on analyzing the randomness of various sources, often neglecting a discussion on the suitability of a specific generator type for particular applications, this article analyzes and compares two types of randomness—pseudorandomness based on a discrete chaotic system and true randomness based on quantum phenomena—and highlights the challenges associated with integrating these solutions into resource-constrained systems. The presented approach provides novel insights into the design trade-offs involved and offers practical guidance for engineers.

## Figures and Tables

**Table 1:** Summary of hardware resources utilized for the IPCore of the logistic map, as presented in Figure 2. The specific gate types used in the implementation are automatically selected during the OpenLane synthesis and place-and-route procedure.

Metric	Value
Core Area [μm^2^]	896,089.4208
CLK Frequency [MHz]	47.61904762
**Number of Cells by Type**	
ANDNOT	9180
AND	423
DFFE PP	45
MUX	48
NAND	533
NOR	5281
NOT	1084
ORNOT	900
OR	3378
XNOR	3479
XOR	6821
**Total Number of Cells**	**31,172**

**Table 2:** NIST randomness test analysis for the logistic map IP Core from Figure 2. Increasing the number of bits used by the NIST package allows for the detection of periodicity associated with the deterministic bitstream generation process based on consecutive $x_{n}$ values in (1). The columns show the proportion of sequences that passed the test. The proportions marked in red indicate values where the NIST suite also reported a potential randomness issue based on the *p*-value. Randomness issues, specifically concerning the *p*-values, were recorded for two versions of the random bit volumes, but their detection did not always apply to the same tests. The results were obtained without applying any post-processing methods acting as randomness extractors.

Test Name	10 × 1,000,000	100 × 1,000,000	10 × 10,000,000
Frequency	10/10	100/100	10/10
BlockFrequency	9/10	97/100	9/10
Cumulative Sums (Forward)	10/10	100/100	10/10
Cumulative Sums (Reverse)	10/10	100/100	10/10
Runs	10/10	99/100	10/10
Longest Run of Ones	10/10	100/100	10/10
Rank	10/10	96/100	10/10
FFT	10/10	100/100	0/10
Non-Overlapping Templates ^+^	1463/1480	9589/9900	1375/1480
Overlapping Templates	10/10	100/100	6/10
Universal	10/10	100/100	10/10
Approximate Entropy	10/10	100/100	0/10
Random Excursions ^+^	79/80	521/528	71/72
Random Excursions Variant ^+^	177/180	1174/1188	160/162
Serial 1	10/10	94/100	0/10
Serial 2	10/10	100/100	0/10
Linear Complexity	10/10	100/100	10/10
	**no issues**	**issues recorded**	**issues recorded**

^+^ Indicates that multiple sub-tests were performed.

**Table 3:** Evaluation of the entropy level and fundamental randomness characteristics for the logistic map IP Core from Figure 2 and for the commercial quantum random number generator from Figure 4 with the usage of ent tool. The randomness disturbances identified by NIST tests do not correspond to a decrease in entropy, which is almost 8 (due to its low sensitivity to randomness disturbances and its more appropriate use in analyzing smaller amounts of data). The only detection of randomness disturbances occurs in the logistic map for a significantly different value in the chi-square test.

Entropy and Basic Randomness Measurements for Logistic Map		
**Test**	**Volume 1**	**Volume 2**
Entropy (bits per byte)	7.999836	7.999656
Optimum Compression rate in %	0	0
Chi-square distribution	283.96 and 10.28%	5959.49 and 0.01%
Arithmetic mean value (127.5 = random)	127.4484	127.4880
Monte Carlo π approximation	3.140875425 (error 0.02 %)	3.137985142 (error 0.11%)
Serial correlation coeff. (totally uncorr. = 0.0)	−0.001300	−0.000630
**Entropy and basic randomness measurements for QUANTIS-USB-4M**		
**Test**	**Volume 1**	**Volume 2**
Entropy (bits per byte)	7.999848	7.999984
Optimum Compression rate in %	0	0
Chi-square distribution	262.54 and 35.94%	269.65 and 25.27%
Arithmetic mean value (127.5 = random)	127.4491	127.4917
Monte Carlo π approximation	3.139838624 (error 0.05%)	3.142840823 (error 0.04%)
Serial correlation coeff. (totally uncorr. = 0.0)	0.000663	−0.000353

**Table 4:** The assessment of the randomness level using NIST statistical tests, conducted for the commercial quantum random number generator QUANTIS-USB-4M from IDQuantique. Regardless of the amount of data generated, the tests show no issues with randomness, which confirms the observed nature of **true randomness**.

Test Name for QUANTIS	10 × 1,000,000	100 × 1,000,000	10 × 10,000,000
Frequency	10/10	99/100	10/10
BlockFrequency	10/10	100/100	9/10
Cumulative Sums (Forward)	10/10	98/100	10/10
Cumulative Sums (Reverse)	10/10	98/100	10/10
Runs	10/10	100/100	9/10
Longest Run of Ones	10/10	98/100	10/10
Rank	10/10	100/100	10/10
FFT	10/10	99/100	10/10
Non-Overlapping Templates ^+^	999/996	9864/9900	788/850
Overlapping Templates	10/10	97/100	10/10
Universal	10/10	99/100	10/10
Approximate Entropy	10/10	99/100	10/10
Random Excursions ^+^	40/40	405/408	63/64
Random Excursions Variant ^+^	90/90	908/912	144/144
Serial 1	10/10	100/100	10/10
Serial 2	10/10	100/100	10/10
Linear Complexity	10/10	99/100	10/10
	**random (no issue)**	**random (no issue)**	**random (no issue)**

^+^ Indicates that multiple sub-tests were performed.

**Table 5:** Examples of threshold values for parameters for the decision tree in Figure 6. The threshold values were chosen for an IoT device that communicates with only one central monitoring unit and sends data at speeds not exceeding 100 MB/s.

Factor	Low	High
Random data volume	from 500 to 50,000	over 50,000
Device size	up to 10 cm × 10 cm × 10 cm	over 10 cm × 10 cm × 10 cm
Budget	up to EUR 1000	over EUR 1000
	**Available**	**Limited**
Reseeding capability	two or more external sensors	no or one external sensor

## Data Availability

The original contributions presented in this study are included in the article. Further inquiries can be directed to the corresponding author. data were created or analyzed in this study. Data sharing is not applicable to this article.

## Abbreviations

Abbreviations used in this manuscript (other abbreviations are explained in text):

ASIC	Application Specific Integrated Circuit
PDK	Process Design Kit
FPGA	Field Programmable Gate Array
NIST	National Institute of Standards and Technology
QRNG	Quantum Random Number Generator
AES	Advanced Encryption Standard
PMT	Photomultiplier Tube
HDL	Hardware Description Language
PCB	Printed Circuit Board
IP Core	Intellectual Property Core
EDA	Electronic Design Automation
CMOS	Complementary Metal Oxide Semiconductor
APD	Avalanche Photo Diode
LED	Light-Emitting Diode
SBC	Single Board Computer
CHSH	Clauser–Horne–Shimony–Holt
SiPM	Silicon Photomultiplier
